# 2-[2,5-Dimeth­oxy-4-(3-nitro­pyridin-2-yl)phen­yl]-3-nitro­pyridine

**DOI:** 10.1107/S2414314625007795

**Published:** 2025-09-11

**Authors:** Mario Geffe, Heiner Detert, Dieter Schollmeyer

**Affiliations:** a University Mainz, Duesbergweg 10-14, 55099 Mainz, Germany; Goethe-Universität Frankfurt, Germany

**Keywords:** crystal structure, heterocycle, conjugation, C—H⋯O contacts

## Abstract

The title compound, C_18_H_14_N_4_O_6_, was prepared in a larger project on condensed heterocycles with a focus on the Cadogan reaction. Extension of this method to multiple Cadogan reactions was explored as a way to larger conjugated systems. A twofold Suzuki reaction on a central diboronic acid and chloro­nitro­pyridine gave the bis­(3-nitro­pyridin-2-yl)benzene.

## Abstract 2

The title compound was prepared in a larger project on condensed heterocycles with a focus on the Cadogan reaction. Extension of this method to multiple Cadogan reactions was explored as a way to larger conjugated systems. A twofold Suzuki reaction on a central diboronic acid and chloro­nitro­pyridine gave the bis­(3-nitro­pyridin-2-yl)benzene.

## Graphical abstract

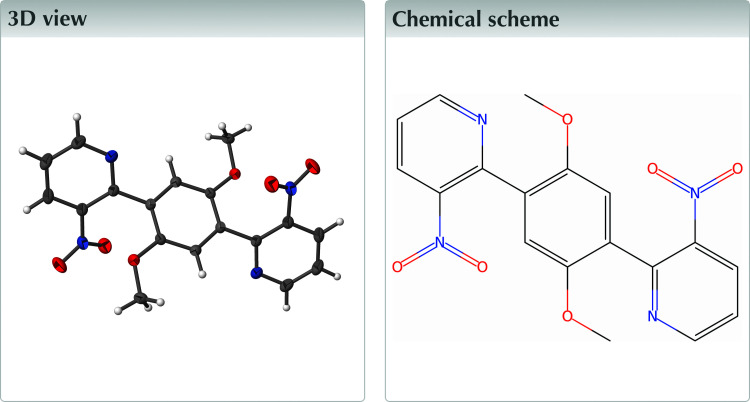

## Structure description

**Figure 1:**
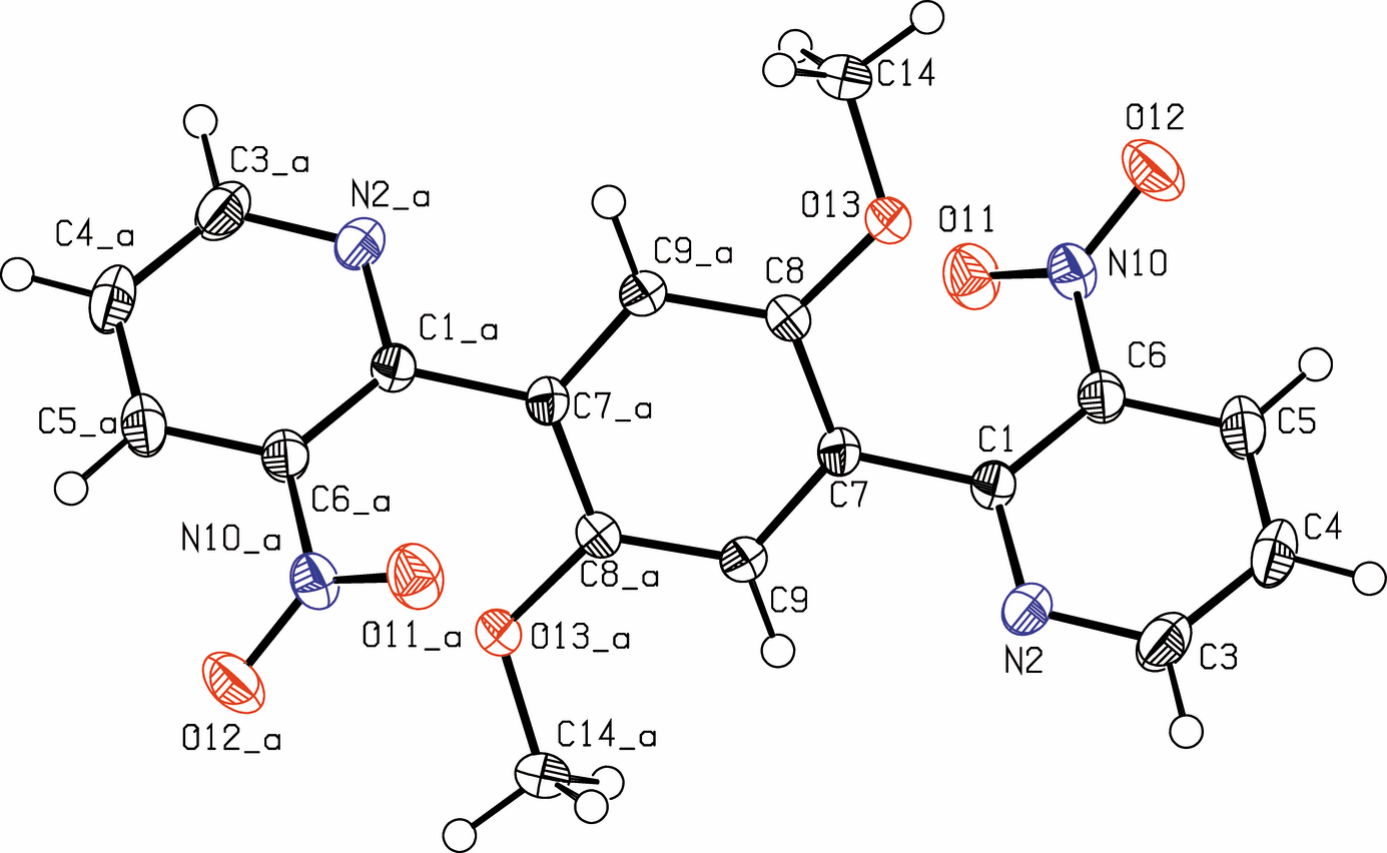
View (Spek, 2009 ▸) of the title compound. Atoms with suffix ‘_a’ were generated using the symmetry operator −*x* + 1, −*y* + 1, −*z* + 1. Displacement ellipsoids are drawn at the 50% probability level.

The title compound, C_18_H_14_N_4_O_6_ (Fig. 1 ▸), was prepared in a larger project on condensed heterocycles (Nissen & Detert, 2011 ▸; Dassonneville *et al.*, 2011 ▸) with a focus on the Cadogan reaction (Letessier *et al.*, 2013 ▸, Limbach *et al.*, 2017 ▸, 2018 ▸). Extension of this method to multiple Cadogan reactions was explored as a way to larger conjugated systems (Wrobel *et al.*, 2012 ▸, 2017 ▸). A twofold Suzuki reaction on a central diboronic acid and chloro­nitro­pyridine gave the bis­(3-nitro­pyridin-2-yl)benzene.

**Figure 2:**
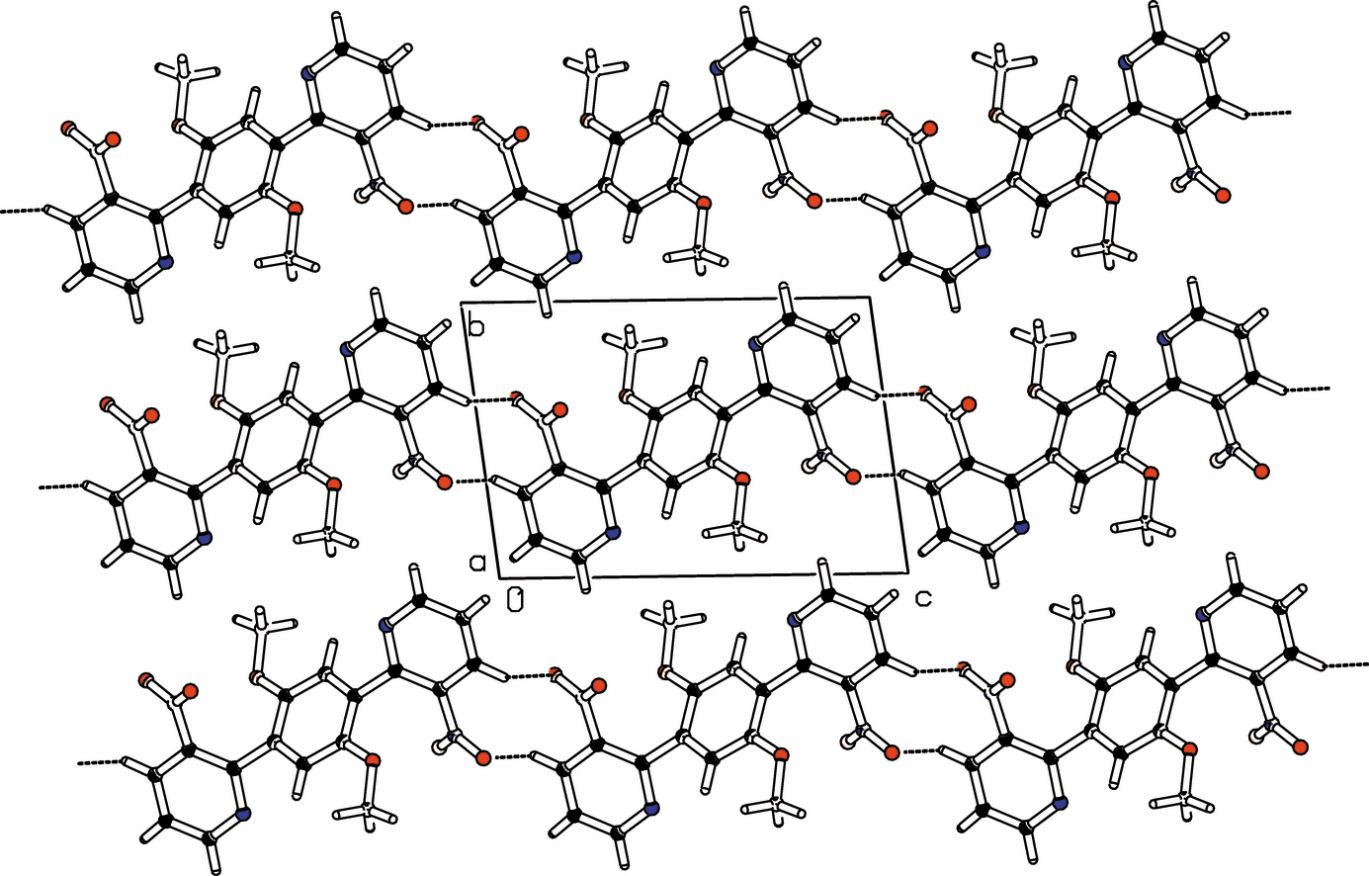
Part of the packing diagram. View along *a*-axis direction (Spek, 2009 ▸). C—H⋯O contacts are drawn as dashed lines.

The unit cell is filled with one centrosymmetric mol­ecule. The mol­ecules form chains in the [10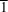] direction, connected *via* hydrogen bridges (H5⋯O12: 2.481 Å) with a C—H⋯O angle of 133.31° (Table 1 ▸). Four substituents in *ortho*-positions of the di­pyridyl­benzene framework provoke torsion angles between the nitro group and the pyridine ring (O11—N10—C6—C1) of −33.41 (16)°, between the pyridine and phenyl­ene (C6—C1—C7—C8) rings of −43.73 (17)° and between the meth­oxy group and the phenyl­ene ring (C14—O13—C8—C7) of 169.47 (10)°. The packing is shown in Fig. 2 ▸.

## Synthesis and crystallization

126.9 mg of 2-chloro-3-nitro­pyridine and 117.4 mg 2,5-di­meth­oxy­phenyl­ene-1,4- diboronic acid, 201.6 mg sodium bicarbonate 1.5 ml water and 1.5 ml 1,2-di­meth­oxy­ethane were mixed in a microwave vessel and the mixture was purged with nitro­gen for 10 min. Tetra­kis-tri­phenyl­phosphine palladium (46.2 mg) was added and the mixture was stirred while microwave irradiation, 100 W, 150°C, max. 10 bar for 15 min. The mixture was filtered through celite, the filter cake was washed with ethyl acetate (75 ml) and the filtrate was washed with brine (20 ml) and dried over MgSO_4_. Purification by chromatography on silica with petroleum ether/ethyl acetate/triethyl amine (1/1/0.025) as eluent (*R*_f_ = 1/5). Recrystallization from acetone gave 48.2 mg (31%) of an orange-red solid with m.p. = 542–544 K. ^1^H-NMR (CDCl_3_, 400 MHz): 8.87 (*dd*, *J* = 4.7 Hz, *J*′ = 1.5 Hz, 2 H, 6-H pyridine), 8.21 (*dd*, *J* = 8.2 Hz, *J*′ = 1.5 Hz, 2 H, 4-H pyridine), 7.44 (*dd*, *J* = 8.1 Hz, *J*′ = 4.7 Hz, 2 H, 5-H pyridine), 7.26 (*s*, 2 H, phenyl­ene), 3.74 (*s*, 6 H, meth­oxy). ^13^C-NMR (CDCl_3_, 75 MHz): 152.44 (2 C, C-6 py), 150.84 (2 C), 1549.74 (2 C), 147.27 (2 C, C-3 py), 131.95 (2 C), 122.67 (2 C), 113.23 (2 C), 55.6 (2 C, OCH_3_). IR (ATR) 3091, 3073, 3016, 2970, 2938, 2839, 2365, 1594, 1555, 1527, 1499, 1467, 1428, 1385, 1353, 1307, 1212, 1173, 1103, 1052, 1024, 862, 819, 802, 770, 717, 677 cm^−1^. HRMS-ESI: found 383.1004, calculated for C_18_H_14_N_4_O_6_: 383.0992.

## Refinement

Crystal data, data collection and structure refinement details are summarized in Table 2 ▸.

## Supplementary Material

Crystal structure: contains datablock(s) I, global. DOI: 10.1107/S2414314625007795/bt4179sup1.cif

Structure factors: contains datablock(s) I. DOI: 10.1107/S2414314625007795/bt4179Isup2.hkl

Supporting information file. DOI: 10.1107/S2414314625007795/bt4179Isup3.cml

CCDC reference: 2484644

Additional supporting information:  crystallographic information; 3D view; checkCIF report

## Figures and Tables

**Table 1:** Hydrogen-bond geometry (Å, °)

*D*—H⋯*A*	*D*—H	H⋯*A*	*D*⋯*A*	*D*—H⋯*A*
C5—H5⋯O12^i^	0.95	2.48	3.2077 (15)	133

Symmetry code: (i) 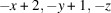.

**Table 2:** Experimental details

Crystal data	
Chemical formula	C_18_H_14_N_4_O_6_
*M*_r_	382.33
Crystal system, space group	Triclinic, *P* 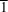
Temperature (K)	193
*a*, *b*, *c* (Å)	4.5590 (4), 8.1530 (8), 11.6359 (10)
α, β, γ (°)	95.552 (7), 95.643 (7), 104.440 (7)
*V* (Å^3^)	413.49 (7)
*Z*	1
Radiation type	Mo *K*α
μ (mm^−1^)	0.12
Crystal size (mm)	0.80 × 0.40 × 0.10

Data collection	
Diffractometer	Stoe *IPDS* 2T
Absorption correction	–
No. of measured, independent and observed [*I* > 2σ(*I*)] reflections	4074, 1948, 1691
*R*_int_	0.020
(sin θ/λ)_max_ (Å^−1^)	0.660

Refinement	
*R*[*F*^2^ > 2σ(*F*^2^)], *wR*(*F*^2^), *S*	0.034, 0.104, 1.08
No. of reflections	1948
No. of parameters	128
H-atom treatment	H-atom parameters constrained
Δρ_max_, Δρ_min_ (e Å^−3^)	0.34, −0.17

Computer programs: *X-AREAWinXpose*, *Recipe* and *Integrate* (Stoe & Cie, 2020 ▸), *SHELXT2014* (Sheldrick, 2015*a* ▸), *SHELXL2019/2* (Sheldrick, 2015*b* ▸) and *PLATON* (Spek, 2009 ▸).

## full crystallographic data

### 2-[2,5-Dimethoxy-4-(3-nitropyridin-2-yl)phenyl]-3-nitropyridine Crystal data

**Table:**

C_18_H_14_N_4_O_6_	*Z* = 1
*M**_r_* = 382.33	*F*(000) = 198
Triclinic, *P*1	*D*_x_ = 1.535 Mg m^−^^3^
*a* = 4.5590 (4) Å	Mo *K*α radiation, λ = 0.71073 Å
*b* = 8.1530 (8) Å	Cell parameters from 7791 reflections
*c* = 11.6359 (10) Å	θ = 2.6–32.3°
α = 95.552 (7)°	µ = 0.12 mm^−^^1^
β = 95.643 (7)°	*T* = 193 K
γ = 104.440 (7)°	Plate, light orange
*V* = 413.49 (7) Å^3^	0.80 × 0.40 × 0.10 mm

### 2-[2,5-Dimethoxy-4-(3-nitropyridin-2-yl)phenyl]-3-nitropyridine Data collection

**Table:**

Stoe IPDS 2T diffractometer	1691 reflections with *I* > 2σ(*I*)
Radiation source: sealed X-ray tube, 12 x 0.4 mm long-fine focus	*R*_int_ = 0.020
Detector resolution: 6.67 pixels mm^-1^	θ_max_ = 28.0°, θ_min_ = 3.0°
rotation method scans	*h* = −5→6
4074 measured reflections	*k* = −10→10
1948 independent reflections	*l* = −15→15

### 2-[2,5-Dimethoxy-4-(3-nitropyridin-2-yl)phenyl]-3-nitropyridine Refinement

**Table:**

Refinement on *F*^2^	Primary atom site location: structure-invariant direct methods
Least-squares matrix: full	Hydrogen site location: inferred from neighbouring sites
*R*[*F*^2^ > 2σ(*F*^2^)] = 0.034	H-atom parameters constrained
*wR*(*F*^2^) = 0.104	*w* = 1/[σ^2^(*F*_o_^2^) + (0.0588*P*)^2^ + 0.0848*P*] where *P* = (*F*_o_^2^ + 2*F*_c_^2^)/3
*S* = 1.08	(Δ/σ)_max_ < 0.001
1948 reflections	Δρ_max_ = 0.34 e Å^−^^3^
128 parameters	Δρ_min_ = −0.17 e Å^−^^3^
0 restraints	

### 2-[2,5-Dimethoxy-4-(3-nitropyridin-2-yl)phenyl]-3-nitropyridine Special details

**Table:**

Geometry. All esds (except the esd in the dihedral angle between two l.s. planes) are estimated using the full covariance matrix. The cell esds are taken into account individually in the estimation of esds in distances, angles and torsion angles; correlations between esds in cell parameters are only used when they are defined by crystal symmetry. An approximate (isotropic) treatment of cell esds is used for estimating esds involving l.s. planes.
Refinement. Hydrogen atoms were placed at calculated positions and were refined in the riding-model approximation with C_aromatic_–H = 0.95 Å or C_methyl_–H = 0.98 Å and with *U*_iso_(H) = 1.2 *U*_eq_(C_aromatic_) or *U*_iso_(H) = 1.5 *U*_eq_(C_aromatic_).

### 2-[2,5-Dimethoxy-4-(3-nitropyridin-2-yl)phenyl]-3-nitropyridine Fractional atomic coordinates and isotropic or equivalent isotropic displacement parameters (Å^2^)

**Table:**

	*x*	*y*	*z*	*U*_iso_*/*U*_eq_	
O11	0.4116 (2)	0.60772 (12)	0.20547 (8)	0.0343 (2)	
O12	0.7851 (2)	0.64907 (13)	0.10149 (9)	0.0394 (3)	
O13	0.94900 (19)	0.65353 (10)	0.37295 (7)	0.0270 (2)	
N2	0.6056 (3)	0.16286 (13)	0.29256 (9)	0.0286 (2)	
N10	0.6193 (2)	0.56385 (14)	0.16322 (8)	0.0268 (2)	
C1	0.6034 (2)	0.32632 (14)	0.28580 (9)	0.0220 (2)	
C3	0.6787 (3)	0.07374 (16)	0.20193 (12)	0.0343 (3)	
H3	0.671276	−0.042903	0.206503	0.041*	
C4	0.7641 (3)	0.14116 (18)	0.10231 (11)	0.0351 (3)	
H4	0.828474	0.075232	0.042713	0.042*	
C5	0.7537 (3)	0.30699 (17)	0.09130 (10)	0.0300 (3)	
H5	0.806523	0.357775	0.023507	0.036*	
C6	0.6635 (3)	0.39632 (14)	0.18267 (9)	0.0236 (2)	
C7	0.5471 (2)	0.41955 (14)	0.39427 (9)	0.0209 (2)	
C8	0.7265 (2)	0.58398 (14)	0.43752 (9)	0.0212 (2)	
C9	0.3237 (2)	0.33665 (14)	0.45786 (9)	0.0219 (2)	
H9	0.204101	0.224369	0.429246	0.026*	
C14	1.1013 (3)	0.82939 (15)	0.40204 (11)	0.0296 (3)	
H14A	0.950412	0.896490	0.402626	0.044*	
H14B	1.238818	0.865537	0.344292	0.044*	
H14C	1.219809	0.847542	0.479277	0.044*	

### 2-[2,5-Dimethoxy-4-(3-nitropyridin-2-yl)phenyl]-3-nitropyridine Atomic displacement parameters (Å^2^)

**Table:**

	*U*^11^	*U*^22^	*U*^33^	*U*^12^	*U*^13^	*U*^23^
O11	0.0367 (5)	0.0389 (5)	0.0347 (5)	0.0189 (4)	0.0120 (4)	0.0094 (4)
O12	0.0466 (6)	0.0406 (5)	0.0376 (5)	0.0127 (4)	0.0191 (4)	0.0193 (4)
O13	0.0301 (4)	0.0225 (4)	0.0257 (4)	−0.0004 (3)	0.0124 (3)	0.0002 (3)
N2	0.0392 (6)	0.0218 (5)	0.0243 (5)	0.0074 (4)	0.0056 (4)	−0.0001 (4)
N10	0.0301 (5)	0.0311 (5)	0.0207 (5)	0.0087 (4)	0.0059 (4)	0.0061 (4)
C1	0.0230 (5)	0.0217 (5)	0.0200 (5)	0.0038 (4)	0.0043 (4)	0.0001 (4)
C3	0.0456 (7)	0.0250 (6)	0.0323 (6)	0.0119 (5)	0.0055 (5)	−0.0038 (5)
C4	0.0415 (7)	0.0369 (7)	0.0278 (6)	0.0142 (6)	0.0088 (5)	−0.0070 (5)
C5	0.0322 (6)	0.0373 (7)	0.0210 (5)	0.0092 (5)	0.0082 (4)	0.0011 (5)
C6	0.0240 (5)	0.0256 (6)	0.0210 (5)	0.0062 (4)	0.0041 (4)	0.0018 (4)
C7	0.0250 (5)	0.0210 (5)	0.0169 (5)	0.0059 (4)	0.0040 (4)	0.0017 (4)
C8	0.0233 (5)	0.0209 (5)	0.0195 (5)	0.0044 (4)	0.0061 (4)	0.0040 (4)
C9	0.0247 (5)	0.0188 (5)	0.0206 (5)	0.0031 (4)	0.0036 (4)	0.0011 (4)
C14	0.0339 (6)	0.0214 (6)	0.0315 (6)	0.0006 (5)	0.0107 (5)	0.0044 (4)

### 2-[2,5-Dimethoxy-4-(3-nitropyridin-2-yl)phenyl]-3-nitropyridine Geometric parameters (Å, º)

**Table:**

O11—N10	1.2218 (13)	C4—C5	1.3824 (19)
O12—N10	1.2293 (13)	C4—H4	0.9500
O13—C8	1.3640 (12)	C5—C6	1.3840 (15)
O13—C14	1.4198 (14)	C5—H5	0.9500
N2—C3	1.3388 (15)	C7—C9	1.3942 (14)
N2—C1	1.3451 (15)	C7—C8	1.4003 (15)
N10—C6	1.4646 (15)	C8—C9^i^	1.3889 (14)
C1—C6	1.4000 (15)	C9—H9	0.9500
C1—C7	1.4872 (14)	C14—H14A	0.9800
C3—C4	1.3794 (19)	C14—H14B	0.9800
C3—H3	0.9500	C14—H14C	0.9800
			
C8—O13—C14	117.88 (9)	C5—C6—N10	116.39 (10)
C3—N2—C1	118.87 (11)	C1—C6—N10	121.94 (9)
O11—N10—O12	123.78 (11)	C9—C7—C8	119.47 (9)
O11—N10—C6	117.99 (10)	C9—C7—C1	118.98 (9)
O12—N10—C6	118.16 (10)	C8—C7—C1	121.42 (9)
N2—C1—C6	119.33 (10)	O13—C8—C9^i^	124.35 (10)
N2—C1—C7	115.02 (10)	O13—C8—C7	115.99 (9)
C6—C1—C7	125.60 (10)	C9^i^—C8—C7	119.64 (10)
N2—C3—C4	123.92 (12)	C8^i^—C9—C7	120.89 (10)
N2—C3—H3	118.0	C8^i^—C9—H9	119.6
C4—C3—H3	118.0	C7—C9—H9	119.6
C3—C4—C5	118.38 (11)	O13—C14—H14A	109.5
C3—C4—H4	120.8	O13—C14—H14B	109.5
C5—C4—H4	120.8	H14A—C14—H14B	109.5
C4—C5—C6	117.56 (11)	O13—C14—H14C	109.5
C4—C5—H5	121.2	H14A—C14—H14C	109.5
C6—C5—H5	121.2	H14B—C14—H14C	109.5
C5—C6—C1	121.60 (11)		
			
C3—N2—C1—C6	2.91 (18)	O12—N10—C6—C1	149.52 (12)
C3—N2—C1—C7	−174.74 (11)	N2—C1—C7—C9	−42.07 (15)
C1—N2—C3—C4	2.5 (2)	C6—C1—C7—C9	140.45 (12)
N2—C3—C4—C5	−4.7 (2)	N2—C1—C7—C8	133.75 (12)
C3—C4—C5—C6	1.3 (2)	C6—C1—C7—C8	−43.73 (17)
C4—C5—C6—C1	4.01 (18)	C14—O13—C8—C9^i^	−12.41 (17)
C4—C5—C6—N10	−172.96 (11)	C14—O13—C8—C7	169.47 (10)
N2—C1—C6—C5	−6.26 (18)	C9—C7—C8—O13	177.20 (10)
C7—C1—C6—C5	171.13 (11)	C1—C7—C8—O13	1.40 (16)
N2—C1—C6—N10	170.55 (10)	C9—C7—C8—C9^i^	−1.02 (18)
C7—C1—C6—N10	−12.07 (18)	C1—C7—C8—C9^i^	−176.82 (10)
O11—N10—C6—C5	143.55 (11)	C8—C7—C9—C8^i^	1.03 (18)
O12—N10—C6—C5	−33.52 (16)	C1—C7—C9—C8^i^	176.93 (10)
O11—N10—C6—C1	−33.41 (16)		

Symmetry code: (i) −*x*+1, −*y*+1, −*z*+1.

### 2-[2,5-Dimethoxy-4-(3-nitropyridin-2-yl)phenyl]-3-nitropyridine Hydrogen-bond geometry (Å, º)

**Table:**

*D*—H···*A*	*D*—H	H···*A*	*D*···*A*	*D*—H···*A*
C5—H5···O12^ii^	0.95	2.48	3.2077 (15)	133

Symmetry code: (ii) −*x*+2, −*y*+1, −*z*.
